# Surface morphology of the oral cavity of redbelly tilapia, Coptodon zillii (Gervais, 1848)

**DOI:** 10.1186/s12917-025-04620-3

**Published:** 2025-03-17

**Authors:** Basma G. Hanafy, Mohamed M. A. Abumandour

**Affiliations:** Department of Anatomy and Embryology, Faculty of Veterinary Medicine, Alexandria University, Abis 10th, P.O. 21944, Alexandria, Egypt

**Keywords:** Dentary teeth, Premaxillary teeth, SEM, Taste buds, *Tilapia zillii*

## Abstract

There is a scarcity of morphological data on the oral cavity of *Tilapia zillii*, so the current investigation aimed to reveal these morphological characteristics, focusing on the teeth of the upper and lower jaws, oral valves, palate, and tongue through both gross anatomical and scanning electron microscopic examinations. The premaxillary and dentary teeth were arranged in rostral and caudal groups with different lengths decreased towards the mouth corners. The rostral group was longer with two processes, while the caudal group had three processes. The rostral group of the premaxillary and dentary teeth was present in one row. While the caudal group of these teeth was present in several rows. At the upper jaw corner, the caudal group was in two rows, while it was in one row at the lower jaw corner. The different lengths in the premaxillary and dentary teeth in conjunction with the presence of processes on the tips of the teeth help shred the eaten particles. The oral cavity structures related to feeding habits in *Tilapia zillii* reflect its herbivorous behaviour. The knowledge of the nature of its oral cavity will help in identifying better methods for feeding in aquaculture.

## Research highlights

The premaxillary and dentary teeth of the *Tilapia zillii* were arranged in rostral and caudal groups with different lengths that decreased towards the mouth corners. The rostral group was long and had two processes, while the caudal group was short with three processes. The medial process of the rostral group is longer than the lateral one.

*Tilapia zillii* had a true tongue with a rounded apex.

Taste buds were observed at different parts of the oral cavity.

## Introduction

The morphological characteristics of a fish’s mouth cavity reveal their structural adaptability to different food types, while their oropharyngeal cavity demonstrates plasticity and structural adaptability, indicating their ability to consume various food particles. Fish adaptations enable them to efficiently capture and process prey, contributing to their success as diverse feeders in aquatic ecosystems [1, 2]. The shape and size of their mouth cavity reveal their feeding behavior and ecological niche. The oral cavity plays a crucial role in food intake and environmental change detection, with oral glands agglutinating food and taste buds playing key functions [1–15].

The morphology of fish feeding apparatus is influenced by various factors such as feeding strategy, environmental conditions, resource utilization, ecological community, structure, and speciation process [1–15]. The previous study examined the mouth and pharyngeal cavities in various fish species, revealing that the mouth cavity is utilized to select desirable food particles or reject undesirable ones, adapted for different food particles in different fish species [1, 11, 16, 17]. Teleost’s oral cavities morphology, tooth presence, and structure vary significantly among species, making it challenging to apply universal descriptions [18]. Understanding this diversity is crucial for understanding feeding behaviors and ecological roles. Future research should explore how these variations impact the fitness and survival of different fish species in their natural environments.

Tilapia, a highly productive and internationally traded food fish, is known for its rapid growth rate and adaptability to different ecological conditions, making it a preferred choice for aquaculture and contributing to the management of freshwater ecosystems by controlling aquatic weeds [19]. *Tilapia zillii* has economic and ecological importance as food fish, for aquaculture, weed control, commercial aquarium trade, and recreational fishery [19]. Tilapia zillii is a versatile fish that thrives in various water quality and environmental conditions, inhabiting lakes, rivers, wetlands, estuaries, and marine habitats. It can tolerate a wide range of salinity levels, making it a popular species for aquaculture worldwide due to its resilience and adaptability [20]. It is one of the *Coptodon zillii* species, order *Perciformes*, family *Cichlid*, and genus *Coptodon*. It is widely distributed in the freshwater and lakes in Africa, especially in Egypt. It is a herbivorous fish that feeds on algae, macrophytes, aquatic insects, and fish eggs [21].

Morphological data are scarce on the oral cavity of *Tilapia zillii*, except that were published about the gills of *Tilapia zillii* [22]. Therefore, this study will be focused on describing the morphological features, with new insights into the teeth of the upper and lower jaws, oral valves, palate, and tongue through gross anatomical and scanning electron microscopic techniques. The study aims to understand the oral morphology of *Tilapia zillii*, enhancing our comprehension of its feeding behavior and ecological niche, and potentially impacting aquaculture practices and conservation efforts of this species. This research can also provide insights into the evolutionary adaptations of *Tilapia zillii* oral structures, revealing its ecological role in aquatic ecosystems. By gaining a better understanding of the species feeding habits, conservation strategies can be better informed to protect this crucial fish species.

## Materials and methods

The present work was conducted on ten mature healthy fish of the *Tilapia zillii* (redbelly tilapia) collected in January from fisher’s shops after catching from Burullus Lake, Kafr El-sheikh governorate, Egypt, their weights ranged between 40 and 60 g. and the total length was 12–16 cm. The samples were transported in a plastic aquarium within 2 h to our anatomical lab to perform the gross anatomical and scanning electron microscopic examinations. All fish were anesthetized using benzocaine (4 mg/L). The collected fish followed the guidelines established for the ‘Sampling protocol for the pilot collection of catch, effort, and biological data in Egypt’ [23]. This study has been carried out with ethical permission from the Faculty of Veterinary Medicine, Alexandria University, and approved by the Institutional Animal Care and Use Committee (**ALEXU-IACUC**) (**Approval code: 262/2023)**.

### For gross anatomical examination

Five samples were dissected and the roofs were separated from the floors by a scissor and washed with physiological saline (0.9% sodium chloride solution) and the oral cavity was photographed by a digital camera Olympus Plus camera (Olympus, Tokyo, Japan).

### For scanning electron microscopic examination

We fixed the samples in a fixed solution of 2% formaldehyde, 1.25% glutaraldehyde, and 0.1 M sodium cacodylate buffer at pH 7.2 and 4 °C for 24 h. Following fixation, the samples were dehydrated through graded series of ethanol and critical point dried and then attached to aluminum stubs facing upwards, covered with carbon tabs, and sputtered with gold. The samples were examined and photographed using a JEOL 5300 ISM Scanning Electron Microscope operating at 25 K.V. at the Faculty of Science at Alexandria University.

## Results

### Gross morphological observations

**Fig. 1:**
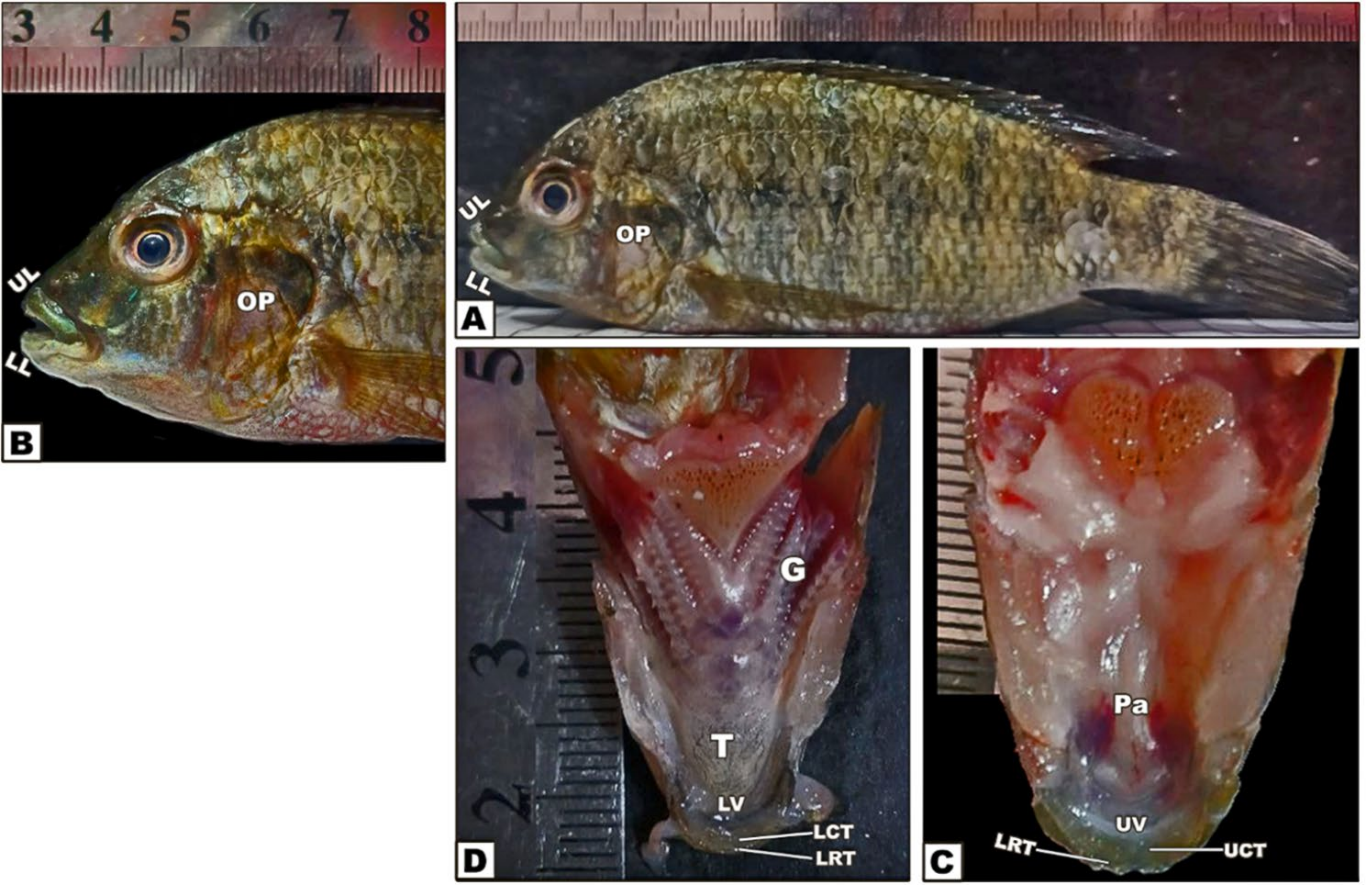
Macrograph shows the gross morphological features of the oral cavity of *Tilapia zillii*. Views (**a** & **b**) show its terminal mouth opening. View (**c**) shows the roof of the oral cavity. View (**d**) shows the floor of the oral cavity. g-gills, LCT- lower caudal teeth, LL- lower lip, LRT- lower rostral teeth, LV- lower valve, OP-gill operculum, Pa- palate, T- tongue, UCT- upper caudal teeth, UL- upper lip, URT- upper rostral teeth, UV- upper valve

The buccal cavity of *Tilapia zillii* had a small, narrow, and terminal mouth opening. The buccal cavity consisted of upper and lower jaws bordered by the lower and upper lips **(**Fig. 1a**)**. The roof of the buccal cavity consisted of the upper jaw, upper semilunar valve, and palate **(**Fig. 1b**)**. The floor of the buccal cavity was composed of the lower jaw, lower semilunar valve, and tongue **(**Fig. 1c**).**

The upper jaw had premaxillary teeth and the lower jaw had dentary teeth. The premaxillary and dentary teeth were present in two groups; the rostral group and the caudal group. The rostral group was long and presented in one row, while the teeth of the caudal group were short and presented in several rows **(**Fig. 1b**/ URT&UCT**, Fig. 1c**/ LRT&LCT)**.

### Scanning electron microscopic observations

#### The roof of the buccal cavity

**Fig. 2:**
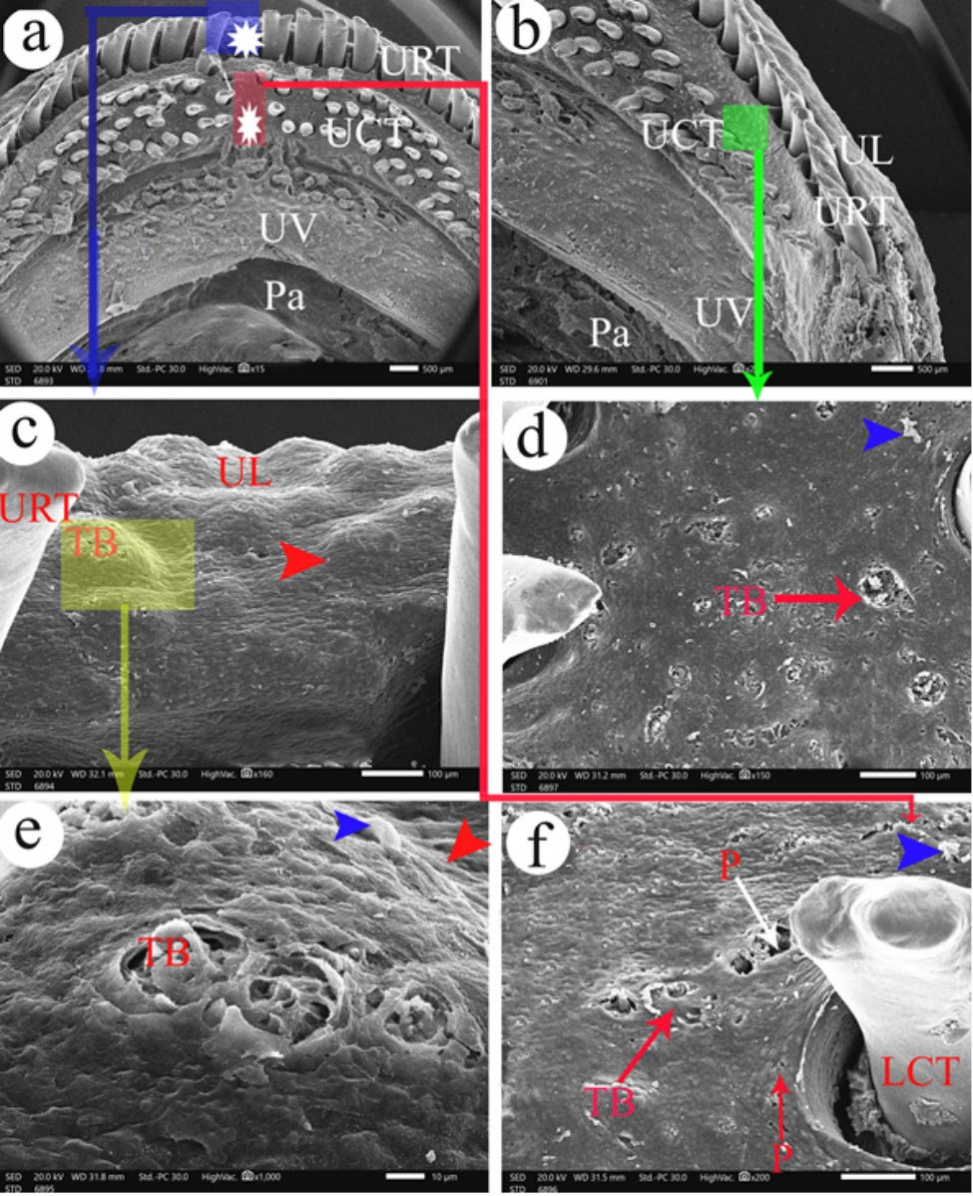
SEM photograph shows the upper lip (UL) and the upper jaw of the *Tilapia zillii*. View (**a**) shows the middle part of the upper jaw. View (**b**) shows the corner of the upper jaw. View (**c**) shows the upper lip. View (**d** and **f**) shows the taste buds between the teeth of the upper jaw. View (**e**) shows the magnification of taste buds on the upper lip. Pa- palate, P- pore of mucous gland, TB- taste buds, UCT- upper caudal teeth, UL- upper lip, UV- upper valve, URT- upper rostral teeth. The red arrowheads refer to the highly elevated epithelial protrusions. The epithelium appeared like fish scales (blue arrowheads). The asterisks refer to the median area that is devoid of the premaxillary teeth

The premaxillary teeth were observed on the upper jaw in two groups: the upper rostral group and the upper caudal group with different lengths that decreased laterally towards the mouth corners. Between the right and left halves of the upper jaw, there was an area devoid of the rostral and caudal groups of premaxillary teeth **(**Fig. 2a**).**

The upper rostral group of the premaxillary teeth was long and presented in one row with two processes on their tips, medial and lateral processes **(**Fig. 2a**/URT).** The medial process of the upper rostral teeth was longer than the lateral one. Moreover, the upper caudal group was present in several rows with three processes on their tips, and the teeth weren’t arranged at the same lines **(**Fig. 2a**/UCT)**. The upper caudal teeth at the corner of the upper jaw were present in two rows only **(**Fig. 2b**/UCT)**.

The area between the rostral and the caudal groups of the premaxillary teeth and the areas between the upper caudal teeth had taste buds at the level of the surface epithelium **(**Fig. 2d**&f/TB)**. The premaxillary teeth were bordered by the upper lip that had taste buds on highly elevated epithelial protrusions **(**Fig. 2c**&e/TB)**.

**Fig. 3:**
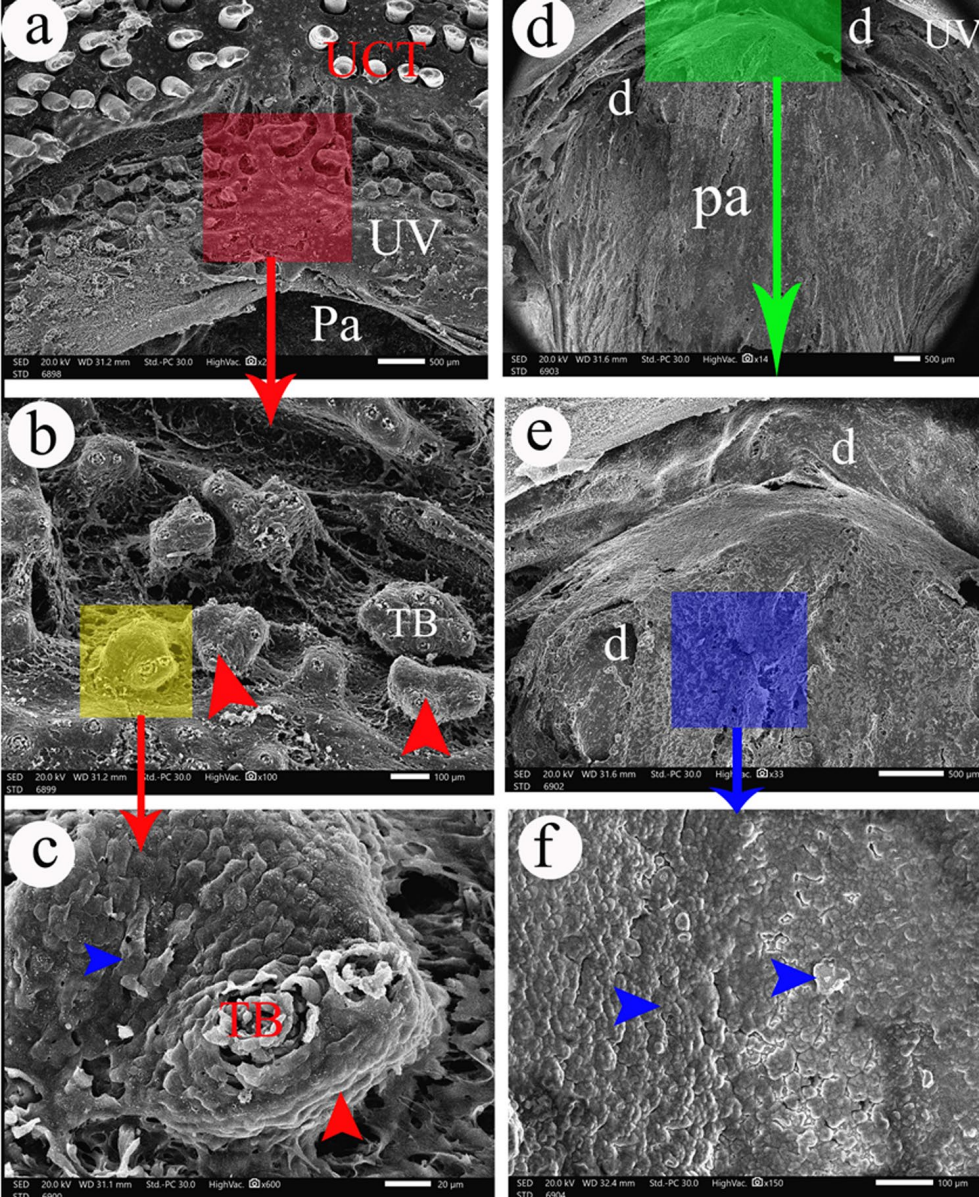
SEM photograph shows the upper valve (UV) in view (**a**) and its magnification in views (**b** and **c**) to show taste buds on highly elevated epithelial protrusions (red arrowhead) and the palate (Pa) in view (**b**) to show the presence of depressions (**d**) and the epithelium appeared like fish scales (blue arrowheads). UCT- upper caudal teeth, UV- upper valve

The upper valve is semilunar in outline, located caudally to the upper caudal premaxillary teeth **(**Fig. 3a/UV), and had taste buds on highly elevated epithelial protrusions **(**Fig. 3b**&c/TB)**. The palate had several depressions, and the epithelium appeared like the fish scales **(**Fig. 3d-f**)**.

#### The floor of the buccal cavity

**Fig. 4:**
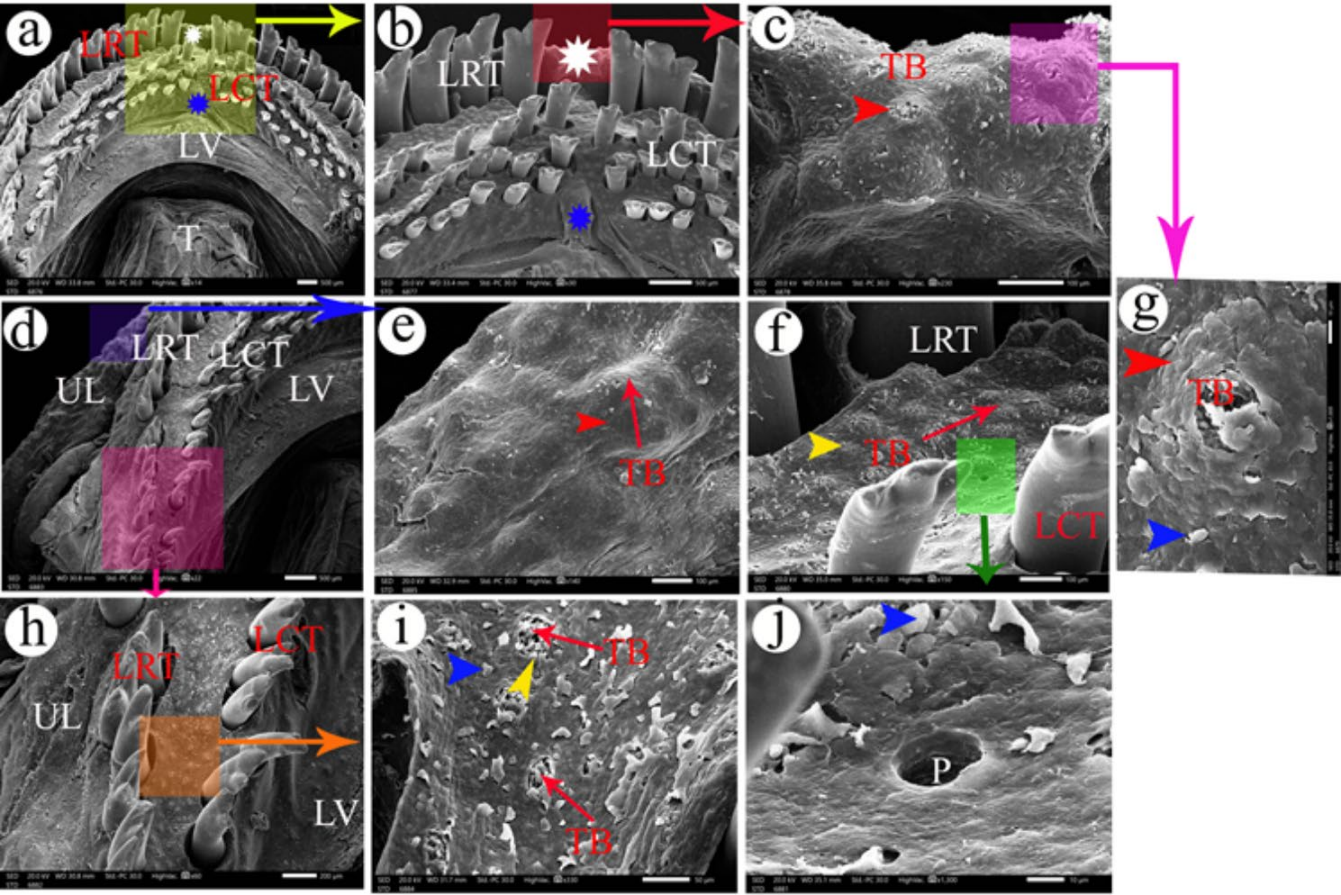
SEM photograph shows the lower jaw and the lower lip. Views (**a** and **b**) show the middle part of the lower jaw. Views (**c** and **e**) show the lower lip. Views (**d** and **h**) show the corner of the lower jaw. Views (**f** and **i**) show taste buds (TB) between the teeth of the lower jaws. View (**j**) shows the pore of the mucous gland (P) and the epithelium appeared like fish scales (blue arrowheads). View (**g**) shows the magnification of taste bud (TB) on highly elevated epithelial protrusion (red arrowhead). LCT- lower caudal teeth, LRT- lower rostral teeth, LV- lower valve, P- pore of mucous gland, T- tongue. The yellow arrowheads refer to slightly elevated epithelial protrusions. The epithelium appeared like fish scales (blue arrowheads). The white asterisks refer to the median area that is devoid of the lower rostral teeth. The blue asterisks refer to a median elevated area between the lower caudal teeth

The dentary teeth were observed on the lower jaw in two groups: the lower rostral group and the lower caudal group with different lengths that decreased laterally. Between the right and left lower rostral groups, there was a median area devoid of these teeth, and between the lower caudal dentary teeth, there was a median elevated area that fit in the area that was devoid of the upper caudal premaxillary teeth **(**Fig. 4a**&b).**

The lower rostral group of the dentary teeth was presented in one row, while the lower caudal group was present in several rows except at the corners of the lower jaw; it was present in one row. The two groups of the dentary had the same appearance as the two groups of the premaxillary teeth **(**Fig. 4d**&h)**. The dentary teeth were bordered by the lower lip, which had taste buds on highly elevated epithelial protrusions **(**Fig. 4c, e **&g/TB)**. The areas between the dentary teeth had taste buds on slightly elevated epithelial protrusions **(**Fig. 4f**/TB)** and taste buds at the level of the surface epithelium **(**Fig. 4i**/ TB)**.

**Fig. 5:**
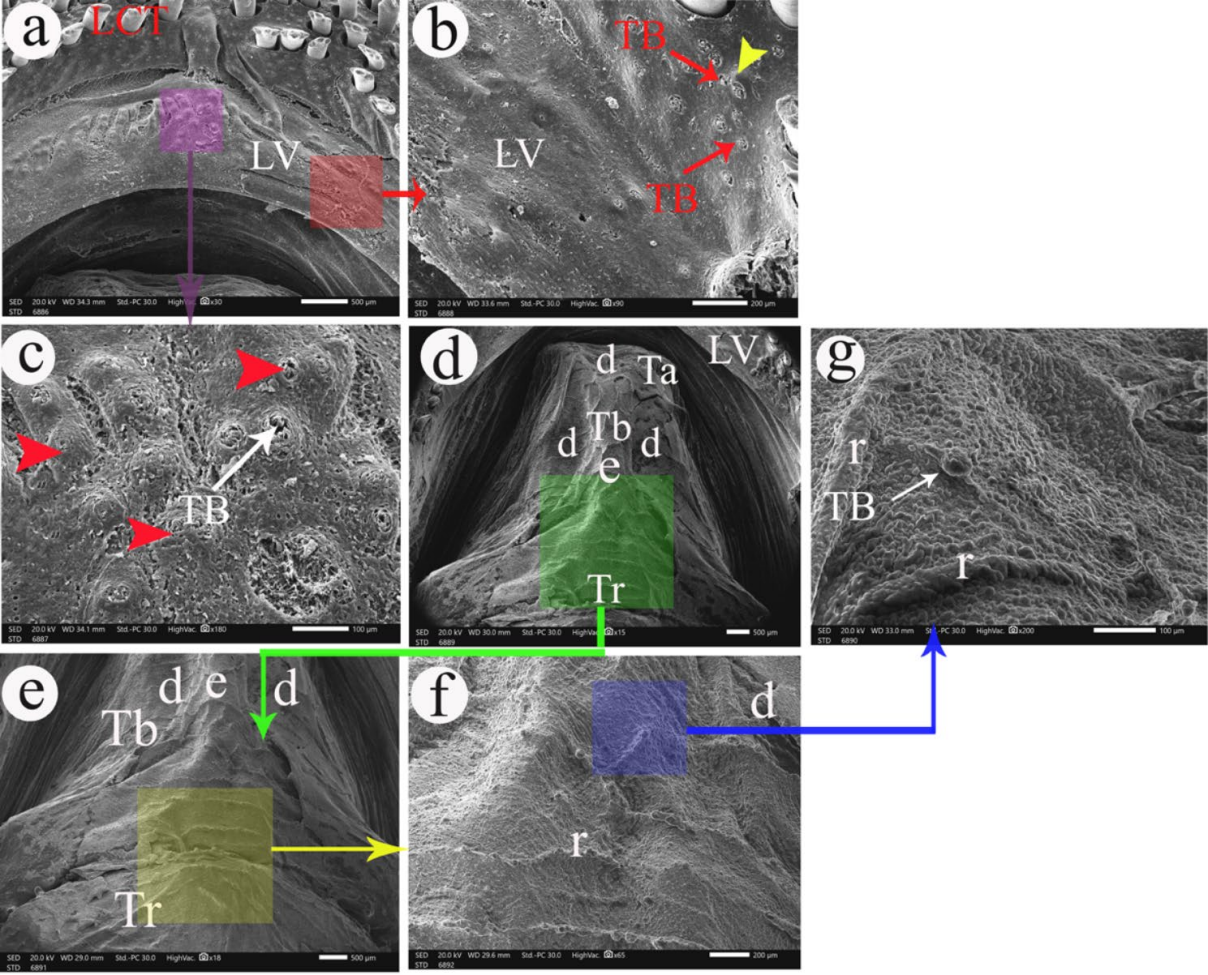
SEM photograph shows the lower valve in views (**a**, **b**, and **c**) and the tongue in views (**d-g**). e- central elevation of the tongue, d- depressions, LCT- lower caudal teeth, LV- lower valve, r-ridges, Ta- Lingual apex, Tb- lingual body, Tr- Lingual root, TB- taste buds. The yellow arrowheads refer to slightly elevated epithelial protrusions. The red arrowheads refer to highly elevated epithelial protrusions

The lower valve was semilunar in outline, located caudally to the lower caudal dentary teeth, and had taste buds on highly elevated epithelial protrusions **(**Fig. 5a**&c/TB)**, taste buds on slightly elevated epithelial protrusions, and taste buds at the level of the surface epithelium **(**Fig. 5b**/TB)**.

The tongue was a true tongue composed of a root, body, and apex. The lingual root was wider than its apex. The lingual apex was rounded with a central depression **(**Fig. 5d**)**. The lingual body had a central elevation with two depressions on its sides **(**Fig. 5d**&e/Tb)**. The lingual surface had several ridges, and it had taste buds on highly elevated epithelial protrusions **(**Fig. 5f**&g)**.

The epithelium of the oral cavity of *Tilapia zillii* appeared like the fish scales **(**Figs. 3c and 4g and i **& j/blue arrowheads)** and it had pores for the mucous glands **(**Fig. 2f **and** Fig. 4j**/P).**

## Discussion

The dentition description, taste buds, mucous cell distribution, and microridge patterns on the oral epithelium along the oral roof and floor of the fish have been recorded by **Alsafy**, et al. [4], **Alsafy**, et al. [5], **Khillare**, et al. [13], **Chanu**, et al. [14], **Sayed**, et al. [15], **Baaoom** [24], **Harabawy**, et al. [25], **Abumandour**, et al. [26], **Abumandour and El-Bakary** [27]. The irregularity and distribution of taste buds and mucous cells, in addition to the microridges patterns of the epithelial surface of the oral roof and floor of *Tilapia zillii*, are considered adaptations to fish feeding behavior and preferences.

The variations in the morphology of the teeth and taste buds, epithelial surface, and mucous cells of the oral cavity of different fish species are considered to be adaptations to different food preferences and feeding habits [7, 28–31].

The present work focused on the morphological features of the oral cavity of the *Tilapia zillii* with concern to the surface epithelium of the upper and lower valves, palate, and the characteristics of the teeth in the lower and upper jaws to give basic information about the architecture of its oral cavity to help in the formulation of the diet necessary for the fisheries of this fish species.

**Khallaf and Alne-na-ei** [21] reported that the *Tilapia zillii* is a herbivorous fish, and the current investigation shows that the premaxillary and the dentary teeth of the *Tilapia zillii* are arranged in two groups: the rostral and caudal groups in the lower and upper jaws. The rostral group is long and has two processes, while the caudal group is short with three processes. So, the different lengths in the premaxillary and dentary teeth in conjunction with the presence of processes on the tips in the rostral and caudal groups of the teeth help to shred the eaten particles like the algae. Additionally, **Bonato**, et al. [32] reported that the fish may be herbivorous, carnivorous, and omnivorous depending on their feeding habits and dentation. Moreover, **Debiais-Thibaud**, et al. [33] recorded the presence of different shapes and types of teeth depending on their diets and feeding habits as well as the gene regulatory network involved in tooth morphogenesis that causes evolution in the shape of the teeth.

The taste buds are observed at different extents on the oral epithelium of *Tilapia zillii*; on highly elevated epithelial protrusions, on slightly elevated epithelial protrusions, and at the same level of the epithelium. These findings come in alignment with those observed by **JAKUBOWSKI and WHITEAR** [34], who recognized the presence of these three types of taste buds depending on their dimensions and the extent of protrusions on the surface epithelium. Furthermore, **Gamal**, et al. [35] reported three types of taste buds; type I is found in relatively high epidermal papillae, type II is mostly found in low epidermal papillae and type III taste buds never rise above the normal epithelial level.

The presence of taste buds in the oral cavity of the fish, including their jaws, oral valves, and tongue, confirms its gustatory ability as it plays a role in the selection of the desirable food particles from the undesirable ones. These results coincide with those obtained by **Hara** [36]; **Kubitza and Lovshin** [37]; **Fishelson**, et al. [38]; **Yashpal**, et al. [7]; **Yashpal**, et al. [8]; **Devitsina**, et al. [39]; **Abbate**, et al. [29].

The current findings reveal that the *Tilapia zillii* has upper and lower oral valves. The oral valves have a role in the regulation of the water in the oral cavity. The same findings are recorded in different fish species [3–5, 26]. While **Yashpal**, et al. [8] in *Cirrhinus mrigala* recorded the presence of the upper valve only, and **Coxon and Davison** [40] reported the presence of the cartilaginous valve in New Zealand hagfish (*Eptatretus cirrhatus*). Functionally, *Tilapia zillii*’s unique adaptation enables it to control water flow through its mouth, aiding in respiration and feeding, but further research is needed to understand its impact on its survival and behaviour.

The current findings reveal that the *Tilapia zillii* has a true tongue consisting of a root, body, and apex. The same results are recorded by **Abbate**, et al. [41] in zebrafish *Danio rerio*, **Abbate**, et al. [29] in gilthead seabream Sparus aurata, **Abbate**, et al. [42] in European sea bass *Dicentrarchus labrax*, **Sadeghinezhad**, et al. [43] in northern pike (*Esox lucius*) and **Alsafy**, et al. [5] in Bagrus bayad. While **Bullock and Bunton** [44], **Genten**, et al. [45], **Abumandour and El-Bakary** [46] described the tongue as a triangular elevated thickening in the epithelium. Additionally, **Genten**, et al. [45] reported it as a thickening of the mucosa. The current findings reveal a rounded lingual apex. The same result was reported in Bagrus bayad [5] and *S. williamsi* [47]. While **Fishelson**, et al. [47] reported it as a spatula in *S. fulva*. Moreover, **Alsafy**, et al. [4] reported a pointed lingual apex in white grouper Epinephelus aeneus.

The surface epithelium in different parts of the oral cavity of *Tilapia zillii* is observed like the fish scales. **Sayed**, et al. [15]; **MITTAL and MITTAL** [48]; **Yashpal**, et al. [7]; **El Bakary** [10] reported that the presence of microridges with different arrangements has a role in several functions such as secretion, absorption, flexibility, and mechanical protection. The microridges are considered structures that protect the oral cavity against physical abrasions during food movement and swallowing in addition to epithelial protection against the abrasion, and this is enhanced by the secretions of mucous cells that lubricate ingested food particles [35]. These microridges are channels for the passage of mucus [10]. Its distribution in the oral cavity reflected the high secretory activity of the epithelium in fish [24, 25].

Several studies described the microridges as finger-like prints covering the oral epithelium. These observations are seen in *D. dentex* [49], in *S. dumerilli* [50], in *Rita rita* [7], in *B. docmak* and *C. gariepinus* [25], in *M. kannume*,* C. auratus*,* B. bynni*, and *S. schall* [24], in *O. niloticus* [51]. Those structures protect the oral mucosa from mechanical trauma, and the secreted mucous forms a lubricated surface for the passage of food [50, 52].

The mucus is secreted by mucous goblet cells or glands located in the oral cavity. The secreted mucus lubricates the oral epithelium and the ingested food particles to help the smooth food passage so the oral epithelium will be protected from the occurrence of mechanical injury as it adapts the oral epithelium to be a lubricated surface and thus will lead to the protection of the epithelium from the abrasion [7, 10, 41, 53, 54]. Also, the mucous can inhibit the proliferation and invasion of pathogenic microorganisms in fish epidermis [51]. The mucous holding on the surface of the cells helps to reserve surface area for stretching or distortion and spreading the mucous outside the goblet cells [1, 8, 10, 48].

## Conclusion

Regarding the herbivorous feeding habit of Tilapia zillii, the morphological characterization of its teeth revealed the presence of rostral and caudal groups of premaxillary and dentary teeth with different lengths and carried processes that help the fish to shred the eaten particles like the algae. Understanding the oral cavity of Tilapia zillii can aid in identifying effective feeding methods in aquaculture. It also offers insights into its evolutionary history and ecological niche, enabling the development of more efficient strategies to optimize fish growth and health.

## Data Availability

The datasets used and/or analyzed during the current study are available from the corresponding author on reasonable request.
